# Topical RM191A gel for chronic peripheral neuropathic pain: randomized, double-blind, placebo-controlled, crossover pilot study

**DOI:** 10.3389/fpain.2026.1772899

**Published:** 2026-03-27

**Authors:** Paul A. Glare, Charles Brooker, Andrew D. Weiss, Daniel J. Costa, Sofia Casbolt, Llewellyn Casbolt

**Affiliations:** 1 Northern Clinical School, Sydney Medical School, University of Sydney, Sydney, NSW, Australia; 2 Pain Management Research Institute, Kolling Institute, St Leonards, NSW, Australia; 3 RR MedSciences Pty Ltd., Sydney, NSW, Australia

**Keywords:** analgesia, crossover study, free radicals, neuropathic pain, randomized controlled trial, superoxide dismutase, topical

## Abstract

**Introduction:**

Current pharmacotherapy options for neuropathic pain (NPP) are limited. RM191A is a novel copper-amino acid complex that mimics superoxide dismutase and has anecdotal evidence for fast-onset analgesia. The aim of this pilot randomized clinical trial (RCT) was to evaluate the safety and efficacy of a topical gel containing RM191A compared with placebo in adults with moderate-to-severe chronic peripheral NPP.

**Materials and methods:**

This single-site study was conducted at an Australian pain clinic. Eligible patients were randomized to receive either active or placebo gel, applied four times daily to the painful site for 3 days, followed by a 4-day washout and crossover to the other gel. Co-primary outcomes were changes in the Numerical Pain Rating Scale (NPRS) scores for average pain from baseline to Days 1 and 3. Secondary outcomes included safety/tolerability and neuropathic pain characteristics.

**Results:**

Twenty-seven participants (14 males, 13 females, mean age 67 ± 15 years) were screened, with 25 proceeding to randomization and 24 completing both treatment periods. No first-order (*p* = 0.816) or second-order carryover effects (*p* = 0.322) were detected. The Day 1 coprimary outcome was not significant (*p* = 0.323), but there was a trend toward one-sided statistical significance for the Day 3 coprimary outcome (*p* = 0.073). The mean reduction in NPRS Average Pain by Day 3 was significant for the active gel (–0.9 ± 1.6, *p* = 0.012) but not the placebo (–0.2 ± 1.7, *p* = 0.485). *Post hoc* analysis revealed the superiority of the active gel in participants with Douleur Neuropathique 4 (DN4) scores ≥4 (*n* = 15, *p* = 0.047). The number needed to treat (NNT) for 50% pain reduction by Day 3 was 8 (95% CI: greater than 3.9). The active gel was more effective against burning pain, paroxysmal pain, and paraesthesia/dysesthesia than other types of NPP. There were no serious adverse events, while four (16%) participants reported mild skin irritation.

**Discussion:**

This small pilot RCT found that 3 days of RM191A gel modestly reduced chronic peripheral neuropathic pain. The NNT of 8 compares favorably with topical lidocaine (14.5) and is similar to duloxetine (7.4) and gabapentinoids (8.9). Larger, longer studies in well-defined neuropathic pain populations are warranted.

**Clinical Trial Registration:**

ANZCTRN 12617000206325. https://www.anzctr.org.au/

## 1 Introduction

Neuropathic pain (NPP) affects approximately 10% of the population and is associated with a higher health impact than non-NPP (1). Treatment of NPP is challenging. Systemic medications such as antidepressants and gabapentinoids are recommended as first-line treatments but provide less-than-satisfactory relief in many cases (2). Topical agents such as capsaicin and lidocaine are second-line options (3); however, the evidence base for them is characterized by modest efficacy and large placebo responses, resulting in number needed to treat (NNT) in the vicinity of 10–15. The limitations of currently available treatments mean novel therapeutic perspectives are urgently required (4).

The search for novel therapies for NPP has focused on the endogenous superoxide dismutase (SOD) system, which protects cells from oxidative stress and inflammation—both implicated in neuropathies and NPP (5, 6). Exogenous SOD is available but has achieved limited development due to its large size (molecular weight ≈ 30 kDa), low cell permeability, short circulating half-life, antigenicity, and high manufacturing costs. To overcome these drawbacks, there has been interest in synthesizing small, stable compounds that mimic SOD's antioxidant activity. Many are under development (7), but none have yet been approved by the US Food and Drug Administration for clinical use.

RM191A is a Cu^3+^-containing coordination complex that mimics SOD but with 10- to 30-fold higher superoxide free radical neutralizing activity (5). RM191A also exhibits potent antioxidant, anti-nitrosative, anti-inflammatory, and immunomodulatory properties *in vitro* and *in vivo (*5). It promotes wound healing and attenuates 12-O-tetradecanoylphorbol-13-acetate (TPA)-induced inflammation and age-associated oxidative stress in mouse skin. It protects human skin explants against UV-induced oxidative stress and DNA damage. It is non-toxic, non-teratogenic, and readily bioavailable in mice (5).

In 2015, a moisturizer containing RM191A came on to the market in Australia. Following its launch, consumers reported consistent symptom relief when it was applied to skin affected by neuropathic pain, indicating unexpected therapeutic efficacy beyond the intended cosmetic use. Recognizing the potential clinical significance of these observations, comprehensive *in vitro* mechanistic studies of RM191A were undertaken. No *in vivo* studies have been undertaken on the cosmetic cream because animal testing of cosmetics has been banned in Australia since 2017. However, due to the reported symptomatic relief, *in vitro* and later *in vivo* studies were conducted on RM191A (not the cosmetic product), which demonstrated biological activity relevant to NPP. Gene expression and cytokine analyses of RM191A revealed significant modulation of key NPP pathways, demonstrating therapeutic potential through both neuroprotective upregulation and anti-inflammatory downregulation pathways (5). In particular, RM191A upregulated heme oxygenase (HMOX-1) and downregulated the WNT5A gene and stromal cell-derived factors 1-alpha and 1-beta (SDF-1a + b) chemokine. It also exerted significant anti-neuroinflammatory effects via downregulation of interleukin-6 (IL-6) and MCP1/CCL2 protein.

Production of the moisturizer was discontinued in 2025 to focus on development of RM191A as a therapeutic agent, a decision not influenced by any regulatory issues or safety concerns. The convergence of spontaneous consumer-reported outcomes and controlled laboratory validation strongly supported the rationale for advancing to a pilot clinical trial, supported by its established safety profile from preexisting cosmetic use and subsequent *in vivo* studies. Therefore, the aim of this pilot study was to formally evaluate the analgesic efficacy of RM191A against chronic peripheral NPP, while also documenting its safety.

## 2 Materials and methods

### 2.1 Trial design

This was a double-blind, placebo-controlled, randomized two-period crossover trial designed to investigate the efficacy, safety, and tolerability of topical RM191A gel compared with placebo gel (vehicle only) in the treatment of chronic peripheral NPP. A crossover design was chosen to make recruitment more efficient as NPP is uncommon in pain clinics, representing only 10%–15% of cases (8). Based on anecdotal reports of the rapid onset of analgesia, we decided to study the effects over 3 days. The washout period was set at 4 days, as negligible carryover effects were expected after topical application.

The study was approved by the Human Research Ethics Committee (HREC) of the Northern Sydney Local Health District (reference number HREC/16/HAWKE/483, dated 1 March 2017). The study was prospectively registered with the Australian Clinical Trial Registry (ACTRN 12617000206325) in February 2017. Reporting followed the CONSORT extension for randomized crossover trials (see Supplementary Appendix for CONSORT Checklist) (9).

Changes to protocol from ANZCTR entry: The final protocol, approved in May 2017, contained some differences compared with the ANZCTR registered 3 months earlier.

- Formulation: RM191A was originally formulated as a spray but was changed to a gel for localized application. Both products had similar ingredients and there was no fundamental difference between them. After four participants were recruited, changes were made to the vehicle to reduce low-grade skin irritation, without altering any other properties of RM191A
- Duration of application: RM191A was applied for 3 days, not 5. The washout period was 4 days, not 3.
- A protocol amendment to change the formulation was approved by the HREC, dated 14 August 2018. Another protocol amendment, dated 27 February 2018, approved a small change in the pharmacy's drug dispensing process.
- Outcomes (including days): the primary outcome was not clearly described in the ANZ Clinical Trial registry. It states, “Change in …NPRS” and then lists the following six time points: “Baseline, Day 1, Day 3, Day 8 (End of Washout), Day 9, Day 11.” In the amended protocol approved by the HREC, the coprimary outcomes were the change from baseline in the mean Numerical Pain Rating Scale (NPRS) for Average pain on Day 1 and Day 3. The stated secondary outcome was the safety of topical application.
- To obtain a better understanding of the activity of RM191A, several other efficacy outcomes were added to the approved protocol, including changes in the NPSI at the same time points as the NPRS.

### 2.2 Participants and setting

### 2.2.1 Participants

Ambulatory adult patients referred by physicians to a hospital-based interdisciplinary outpatient pain clinic were screened. Patients with a medical history suggesting a disease or lesion of the peripheral nervous system who complained of chronic pain in the associated anatomical distribution were approached to enter the study.

#### 2.2.2 Eligibility criteria

##### 2.2.2.1 Inclusion criteria

- Male/female patient aged 18 years or older;
- Moderate-to-severe NPP present for at least 3 months;
- Affected area is appropriate for topical treatment and skin is intact over the area to be treated;
- NPRS for “average pain over the last 24 h” is greater than or equal to 4/10 on at least 3 of the 7 days prior to the baseline visit;
- Prior pain medications were stable for 3 weeks prior to study and expected to be continued at current doses for the duration of the study;
- Women of childbearing potential required a negative pregnancy test and use of highly effective birth control for 1 month before, during, and 1 month after the study; and
- Ability to provide informed consent, complete study diary, and follow protocol requirements.

##### 2.2.2.2 Exclusion criteria

• Significant pain due to causes other than NPP;

- Change in dose of long-acting opioids or adjuvant analgesics within 7 days of the screening visit, during the screening period prior to baseline assessment of pain, and/or during the study;
- Current or recent use of any topically applied non-opioid pain medications on the affected areas within 3 days prior to baseline assessment of pain and during the study;
- History of a dermatological condition or recurrent generalized skin disorder in the area to be treated within last 5 years, including psoriasis, eczema, or any other skin condition that might interfere with study assessments;
- Known allergy to copper;
- Diagnosis of Wilson's Disease;
- Other significant medical comorbidities; and
- Participation in another investigational drug study within 30 days prior to the screening visit.

#### 2.2.3 Setting

The study was conducted at an academic multidisciplinary ambulatory pain center in Sydney, Australia.

### 2.3 Interventions (study treatments)

The active gel consisted of RM191A (2%) and a water-based vehicle (98%) containing deionized water (83.04%), Exilva M 10% microfibrillated cellulose (2.50%), PEG-120 jojoba (2.50%), glycerin 99.5% (1.50%), Euxyl PE 9010 (phenoxyethanol and ethylhexylglycerin, 0.25%), Tween 20 (0.20%), and lavender essential oil (0.01%). Each pump dispensed 0.2 mL of gel, containing 20 mg of RM191A (0.92 mg elemental copper). The gel was applied four times a day for 3 days (at 8 a.m., 12 p.m., 4 p.m., 8 p.m.), with the dosing frequency based on anecdotal reports by consumers of the moisturizer. Up to eight pumps (1.6 mL) of gel could be applied per site, enough to cover approximately 0.5 m^2^ equivalent to the foot plus leg up to the calf. Participants with more extensive pain were instructed not to exceed an area greater than 0.5 m^2^ and to apply it preferentially to the area of worst pain. The placebo gel was identical to the active gel but lacked the active ingredient. Both gels were produced under Good Manufacturing Practices standards at a local facility approved by Australia's Therapeutic Goods Administration (Delta Laboratories Pty Ltd., Somersby, NSW, Australia). The gel dispensers for the active and placebo gels were also identical.

After the first three participants had been treated, minor adjustments were made to the vehicle to improve its application on the skin. This included removal of micro-fibrillated cellulose to reduce dermatological irritation.

### 2.4 Study procedures

**Figure 1:**
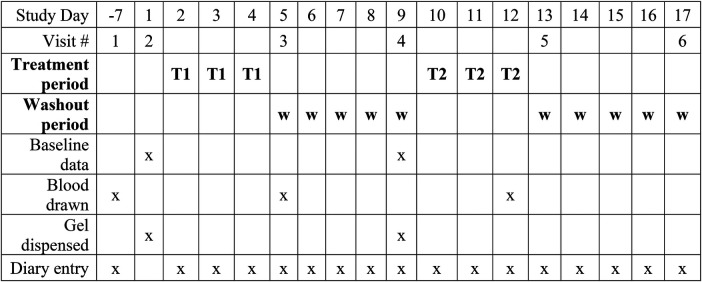
Schedule of activities during the study.

Potential participants were approached by clinic physicians or recruited via advertisements. Those who expressed interest met with a research assistant to provide informed consent and schedule a screening visit. The schedule of activities during the study is shown in Figure 1.

- Screening Visit (Study Visit 1): After confirming eligibility, participants underwent a physical examination, including a focused neurological examination (mental status, motor strength, reflexes, sensory assessment). The IASP grading system terminology (unlikely, possible, probable, definite) was used to classify neuropathic pain (10), with the following modifications: Warm temperature was not tested; allodynia and hyperesthesia were accepted as indicators of neuropathy; like surgery, receiving chemotherapy was considered confirmatory; and electrodiagnostic studies were only recorded if mentioned in the referring physician's letter or documented in the hospital medical records. Baseline pain medications were recorded. Participants received a diary to track study measures, adverse events (AEs), and concomitant medications. During the 7-day screening period, participants recorded average pain scores over the past 24 h at 8 p.m. each evening. At the end of 7 days, participants were contacted by phone and those with at least three consecutive average pain scores of 4–9 were invited to enter the trial, with the baseline visit scheduled for the next business day.
- Baseline Visit (Visit 2, Day 1 of study): Participants were assigned a study ID number and completed baseline assessments, including their average and worst pain scores over the past 24 h, which were used for baseline values for outcome assessments.
  Participants were then randomized in a 1:1 ratio to receive either the active treatment or placebo first. They were given a plain gel dispenser and instructed to begin applying the gel at 8 am the next morning (Day 2). Participants were instructed to record 24-hour average and worst pain (NPRS), concomitant medications, and adverse events in the diary, beginning the evening of Day 2 and continuing through to study completion.
- End of Treatment Period 1 Visit (Visit 3, Day 5): After completing 3 days of gel application at 8 p.m. the previous evening, participants returned for efficacy and safety assessments and diary compliance review. A 4-day washout period followed, with participants continuing their diary entries each evening.
- Treatment Period 2 Visit (Visit 4, Day 9): At the end of the washout period, participants returned for baseline pain and other assessments before beginning Treatment Period 2. Participants were given a dispenser of the alternate gel and began treatment at the same body site as Treatment Period 1, at 8 a.m. the following morning (Day 10).
- End of Treatment Period 2 Visit (Visit 5, Day 13): After completing the 3 days of Treatment Period 2 at 8 p.m. the previous night, participants returned for efficacy and safety assessments and diary review. A second 4-day posttreatment washout period followed, with daily diary entries.
- End-of-Treatment Visit (Visit 6, Day 17): Vital signs, physical and neurological assessments, and safety evaluations were performed. Study measures were completed, and diaries returned. Adverse events (solicited and unsolicited) were recorded.

### 2.5 Outcomes

#### 2.5.1 Prespecified coprimary outcome measures

The coprimary outcomes were changes in NPRS score for average pain from baseline visit to Day 1 and Day 3 for each treatment (active gel and placebo). Numbers NNT for 30% and 50% reductions in average pain score at baseline by Day 1 and Day 3 were calculated.

#### 2.5.2 Secondary outcomes

Given that this was the first clinical study of the gel, its tolerability and safety were important secondary outcomes. Tolerability was evaluated by evidence of skin irritation. Safety was assessed by any clinically significant changes in physical or neurological examination, laboratory test results, or reports of adverse events (AEs).

#### 2.5.3 Exploratory, *post hoc* outcomes

Given the exploratory nature of the pilot study, a limited number of *post hoc* analyses were conducted to better understand the potential characteristics of responders and guide future studies. The following are two secondary efficacy endpoints that were not included in the ANZCTR or approved protocol but pre-specified prior to performing the analysis:

- change from baseline of “worst pain” score on NPRS at Day 1 and 3,
- change in NPP Symptom Inventory (NPSI) scores from baseline to Day 3.

There were no changes to any of the trial outcome measures after the trial commenced.

### 2.6 Outcome measures

#### 2.6.1 NPRS

The NPRS is an 11-point self-report scale measuring pain over the last 24 h, ranging from 0 (no pain) to 10 (worst pain). A two-point reduction is considered clinically significant and recommended for NPP trials (11, 12). A 30%–50% decrease in NPRS is used to calculate the number NNT for pharmacotherapy (13).

#### 2.6.2 NPSI

The NPSI is a validated 12-item scale assessing NPP symptoms (14). Ten items measure pain intensity across five dimensions: evoked, pressing (deep) spontaneous, paroxysmal, paraesthesia/dysesthesia, and burning (superficial) spontaneous pain. Scores range from 0 (no pain) to 100 (most intense pain). Two additional items track the duration of ongoing spontaneous and paroxysmal pain over 24 h. The NPSI is sensitive to treatment changes and can predict treatment outcomes (12).

#### 2.6.3 Douleur neuropathique 4 (DN4)

The DN4 is a 10-item questionnaire used to screen for and assess the likelihood of NPP (15). The questionnaire consists of seven questions about the patient's pain characteristics (like burning or electric shocks) and three questions about clinical findings in the painful area (like numbness or reduced touch sensitivity). A score of 4 or higher out of a possible 10 indicates that NPP is likely, and further clinical evaluation is recommended (16).

#### 2.6.4 Tolerability and safety

A Data Safety Monitoring Board was not established due to the expected safety of the intervention. Skin irritation was an adverse event of interest and was assessed on the 7-item Skin Irritation Scoring scale, based on erythema, edema, papules, or vesicular eruption (16). AEs were assessed through patient reports or phone interviews on Day 3 and at Study Visits 2–6, using the Medical Dictionary for Regulatory Activities (version 7.0). Safety was further evaluated through vital signs, physical exams, and lab results. Elevations of serum copper above the reference range (11.8–22.8 µmol/L) after treatment were considered AEs of interest.

### 2.7 Sample size

Using the Sealed Envelope (https://www.sealedenvelope.com) power calculator for a continuous outcome superiority trial, 46 evaluable patients (23 patients per treatment sequence) were required to detect a mean difference of 1.5 points in NPRS after 3 days between the active arm and placebo arm, using a standard deviation of 1.8 with a power of 80% and a significance of 0.05. To halve the number of participants needed, a crossover design was adopted. Allowing for a 10% screen failure rate, it was estimated that 25 patients would need to be screened. A reduction of 1.5 points in NPRS has been established as the minimal clinically important difference (MCID) for pain (17), while the standard deviation for average NPP has been reported as 1.6–1.9 (18).

### 2.8 Randomization

Randomization, allocation concealment, and blinding were managed by the Sponsor's Clinical Research Organization (CRO) delegate, Mobius Medical Pty. Ltd. (North Sydney, Australia). Participants were randomly allocated in a 1:1 ratio to receive either topical RM191A gel in Treatment Period 1 followed by placebo gel in Treatment Period 2, or placebo gel in Treatment Period 1 followed by topical RM191A gel in Treatment Period 2. A randomization blocking scheme was used to ensure that balance between the treatment groups was maintained. The randomization code was held by the Sponsor's CRO to ensure allocation concealment.

Dispensing and product accountability was by the hospital's Clinical Trials Pharmacy, as per standard regulations for supply of a clinical trial medication. A research assistant contacted the CRO, which provided a code number to the hospital pharmacy for dispensing the study medications. In this manner, the participants, researchers, and clinicians were fully blinded to the treatment order.

### 2.9 Statistical analysis

Changes from baseline to all measured and relevant time points were calculated. The baseline values were those recorded at the planned visits (on Day 2 and Day 9) prior to the first dose in each treatment period. The data were analyzed using the method for 2 × 2 designs with baselines for each condition included, as described by Jones and Kenward (19). In brief, this method involved testing for first- and second-order carryover effects, followed by testing for a direct treatment effect. In the absence of carryover effects, the direct treatment effect was analyzed by comparing two posttreatment time points. Contrast scores were calculated corresponding to each of these three effects, and three independent samples *t*-tests were conducted (one for each effect) with sequence (placebo–active vs. active–placebo) as the independent variable. The data were analyzed using SPSS v26.

Binary outcomes, including absolute risk reduction (ARR) and numbers needed to treat, were calculated for average NPRS. Ancillary * post hoc* analyses were conducted for outcomes in patients who screened positive on DN4 (score ≥4).

## 3 Results

**Figure 2:**
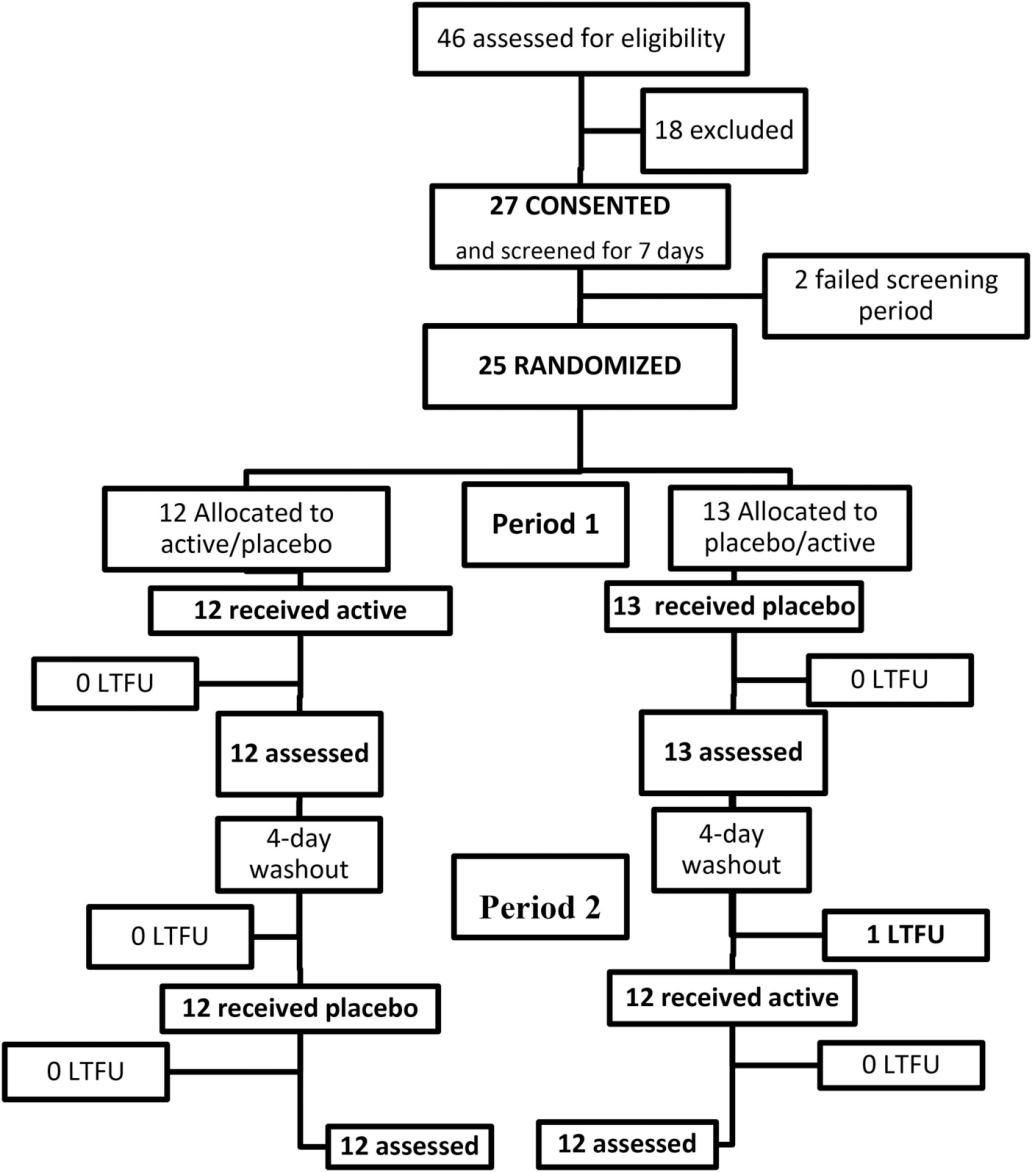
Participant flow diagram. LTFU, lost to follow up.

Participant flow (see Figure 2): A total of 27 participants were randomly assigned and 25 completed screening. Of the two who did not complete the screening phase, one with chemotherapy neuropathy became medically unwell and withdrew; the other had possible NPP and did not meet the inclusion criterion of 3 consecutive days of pain of at least 4 on the NPRS.

The remaining 25 participants completing the screening were randomized. No one dropped out after randomization; however, one dropped out after Treatment Period 1, leaving 24 who received the intended treatment for both periods.

Recruitment and trial end: Recruitment began in September 2017. The trial was planned to end once 22 participants completed the study and was expected to last approximately 6–12 months. However, the recruitment target was reached more than 3 years later, in May 2021. While the 22nd randomized patient was enrolled, two additional patients were recruited and went on to complete the study, bringing the sample size to 24.

### 3.1 Baseline participant data of consented patients collected at the screening visit (*n* = 27)

Baseline characteristics are shown in Table 1. Participants were older than 65 years on average, with similar numbers of males and females. All used neuropathic descriptors to describe their pain. The certainty of their diagnosis of peripheral neuropathic pain is shown in Table 2. All had suggestive diagnoses of damage to peripheral nerves. These diagnoses were heterogeneous, although two-thirds had peripheral neuropathies. All experienced pain in the relevant anatomical distribution. All but one had sensory changes; the exception was an individual with diabetes and painful feet who had normal sensation but reduced reflexes on the exam and abnormal nerve conduction studies. Only a few had neurodiagnostic studies documented at the time of enrollment.

**Table 1:** Clinical characteristics of the 27 eligible patients.

Parameter	Value
Average age (years)	66.7 ± 14.6
Gender	14 (52%) M
	13 (48%) F
Suggested neurological lesion or disease, *n* (%)	
Diabetic neuropathy	5 (15%)
Chemotherapy-induced peripheral neuropathy	3 (19%)
Peripheral neuropathy induced by other drugs	3 (15%)
Peripheral neuropathy, other	7 (41%)
Peripheral nerve injury	
Surgical	6 (11%)
Traumatic	1 (7%)
Current medications (at randomization, *n* = 25)	20 (80%)
Gabapentinoids	7 (28%)
Anti-depressants	3 (12%)
Both Gabapentinoids plus antidepressants	5 (20%)
Opioids	9 (36%)
Non-steroidal anti-inflammatories or acetaminophen	12 (41%)

**Table 2:** Certainty of diagnosis of neuropathic pain.

SID	Suggested neurological diagnosis	Site of pain	Sensory changes	Confirmation of diagnosis	Classification
Randomized					
2	Thoracic PNI postpacemaker insertion	Trunk	Increased	Surgery	Certain
3	Thoracic PNI postcancer surgery	Trunk	Increased	Surgery	Certain
4	PN associated with mitochondrial disease	Feet	Reduced	NCS	Certain
24	Diabetic neuropathy	Feet	Normal sensation but absent reflexes	NCS	Certain
13	PNI from mastectomy	Chest/arm	Increased	Surgery	Certain
20	Plantar PNI postsurgery	Foot	Increased	Surgery	Certain
22	Thoracic PNI post-cancer surgery	Abdominal wall	Reduced	Surgery	Certain
14	Chemotherapy neuropathy	Below knees	Reduced	Postchemo	Certain
16	Chemotherapy neuropathy	Forearms	Reduced	Postchemo	Certain
19	Chemotherapy neuropathy	Feet	Reduced	Postchemo	Certain
21	PN associated with acetazolamide	Below knees	Increased	(Normal NCS)	Probable
7	Small fiber neuropathy	Below knees	Reduced	(Normal NCS)	Probable
1	Postherpetic neuralgia	Trunk	Mixed	Not done	Probable
5	Diabetic neuropathy	Below knees	Reduced	Not recorded	Probable
6	Diabetic neuropathy	Feet	Reduced	Not recorded	Probable
8	Diabetic neuropathy	Feet	Reduced	Not recorded	Probable
9	PN NOS	Below knees	Mixed	Not recorded	Probable
11	PN NOS	Feet	Reduced	Not recorded	Probable
12	SFN associated with erythromelalgia	Feet	Increased	(refused NCS)	Probable
15	PN associated with statin use	Feet	Reduced	Not recorded	Probable
17	PN NOS	Below knees	Reduced	Not recorded	Probable
18	Plantar PNI post-trauma	Foot	Increased	Not recorded	Probable
25	Thoracic PNI post-trauma	Trunk	Increased	Not recorded	Probable
26	Diabetic neuropathy	Below knees	Reduced	Not recorded	Probable
27	PN associated with cephalexin	Below knees	Increased	Not recorded	Probable
Screened. not randomized					
10	Chemotherapy neuropathy	Feet	Reduced	Postchemo	Certain
23	PN NOS	Below knees	Normal	Not recorded	Possible

NCS, nerve conduction studies; NOS, not otherwise specified; PN, peripheral neuropathy; PNI, peripheral nerve injury.

### 3.2 Baseline pain scores of randomized patients (*n* = 25)

The baseline scores for the 25 randomized participants at Visit 2 (prior to Treatment Period 1) are shown in Table 3. Eighty percent were using pharmacotherapy for pain relief, taking a median of 2 analgesic drug classes (range 0–4). Fifteen (60%) were on gabapentinoids and/or antidepressants, all for more than 3 months, with the median of 1 year. Nine (33%) were on opioids, for a median 1.8 years, with two having started them in the past 4–12 weeks.

**Table 3:** Baseline values recorded at visit 2 for the 25 randomized patients.

Questionnaire	Score (average ± standard deviation, *n* = 25)
NPRS (average pain)	6.16 ± 1.40
NPRS (worst pain)	7.88 ± 1.02
NPSI, total score	27 ± 19
DN4	5.5 ± 2.7

#### 3.2.1 Baseline NPRS for average pain (primary outcome) and worst pain (secondary outcome)

Average pain: The overall mean score for average pain at Visit 2 (the baseline visit for Treatment Period 1) was 6.2 ± 1.4. The mean average pain score at Visit 4 (the baseline visit for Treatment Period 2) was similar (6.1 ± 1.3). The difference in mean score of those receiving active gel in Treatment Period 1 (6.6 ± 1.3) versus those receiving the placebo gel (5.8 ± 1.4) was not significant (*t* = 1.52, *p* = 0.143).

Worst pain: The overall mean worst pain score before Treatment Period 1 was 7.9 ± 1.1, and the difference in the worst pain between those receiving active gel first (8.0 ± 0.9) and placebo first (7.8 ± 1.2) was not significant (*p* = 0.64). The worst pain score before Treatment Period 1 was 7.9 ± 1.1 which was higher than that before Treatment Period 2 (7.1 ± 1.7), and this difference approached significance (*t* = 0.196, *p* = 0.055).

#### 3.3.2 NPSI

The average total NPSI score at the baseline visit was 27 ± 19. Sensations of burning, pressing, paroxysmal, and evoked pain were reported by 17, 12, 19, and 18 participants, respectively, and paresthesia or dysesthesia was reported by 17. Burning was a prominent feature with the median score 7, while the median for the other symptoms was ≤4.

#### 3.3.3 Plasma copper level

The average serum copper level at baseline was 16.1 ± 3.2 µmol/L. Two participants had elevated copper levels at baseline but were allowed to continue in the study as these abnormalities were not considered to be clinically relevant. The elevation was mild (<25 µmol/L), they were asymptomatic, and they had normal liver and renal function.

### 3.3 Outcomes and precision estimates

The results of all treatment effect analyses are shown in Table 4.

**Table 4:** Paired t-test results to evaluate for carryover effects and direct treatment effects for NPRS and NPSI scores.

Questionnaire score		First-order carryover	Second-order carryover	Direct treatment effect
NPRS Average Pain	Day 1	*t*(22) = −0.46, *p* = 0.649	*t*(22) = −0.59, *p* = 0.561	*t*(22) = −1.01, *p* = 0.323
	Day 3	*t(22)* *=* *−0.24, p* *=* *0.816*	*t(22)* *=* *−1.01, p* *=* *0.322*	*t(22)* *=* *1.51*, *p* *=* *0.146*
NPRS Worst Pain	Day 3	*t*(11) = 1.24, *p* = 0.241	*t*(11) = 1.00, *p* = 0.339	*t*(20) = 1.84, *p* = 0.081
Total NPSI	Day 3	*t*(22) = 1.01, *p* = 0.324	*t*(22) = −021, *p* = 0.832	*t*(22) = −1.32, *p* = 0.199
NPSI burning	Day 3	*t*(22) = 1.27, *p* = 0.217	*t*(22) = 0.71, *p* = 0.486	*t*(22) = −1.92, *p* = 0.068
NPSI brushing (one item)	Day 3	*t*(20) = 0.50, *p* = 0.622	*t*(20) = 0.34, *p* = 0.740	*t*(20) = 1.94, *p* = 0.067
NPSI pins and needles (1 item)	Day 3	*t*(20) = 0.41, *p* = 0.689	*t*(20) = 0.85, *p* = 0.408	*t*(20) = 1.55, *p* = 0.136
NPSI pressing	Day 3	*t*(22) = 1.61, *p* = 0.121	*t*(22) = 0.44, *p* = 0.667	*t*(20) = −0.71, *p* = 0.484
NPSI paroxysmal	Day 3	*t*(20) = −0.52, *p* = 0.605	*t*(22) = 0.03, *p* = 0.975	*t*(20) = −0.20, *p* = 0.846
NPSI evoked	Day 3	*t*(22) = 0.64, *p* = 0.527	*t*(22) = 0.27, *p* = 0.787	*t*(22) = −0.67, *p* = 0.508
NPSI para/dysesthesia	Day 3	*t*(22) = 1.95, *p* = 0.064	*t*(22) = −0.44, *p* = 0.666	*t*(22) = −0.79, *p* = 0.436

All 25 randomized patients received the placebo gel and 24 received the active gel. All analyses were done according to the original assigned groups.

#### 3.3.1 Coprimary outcomes: direct treatment effect for change in average pain NPRS score from baseline to Day 1 and Day 3

**Figure 3:**
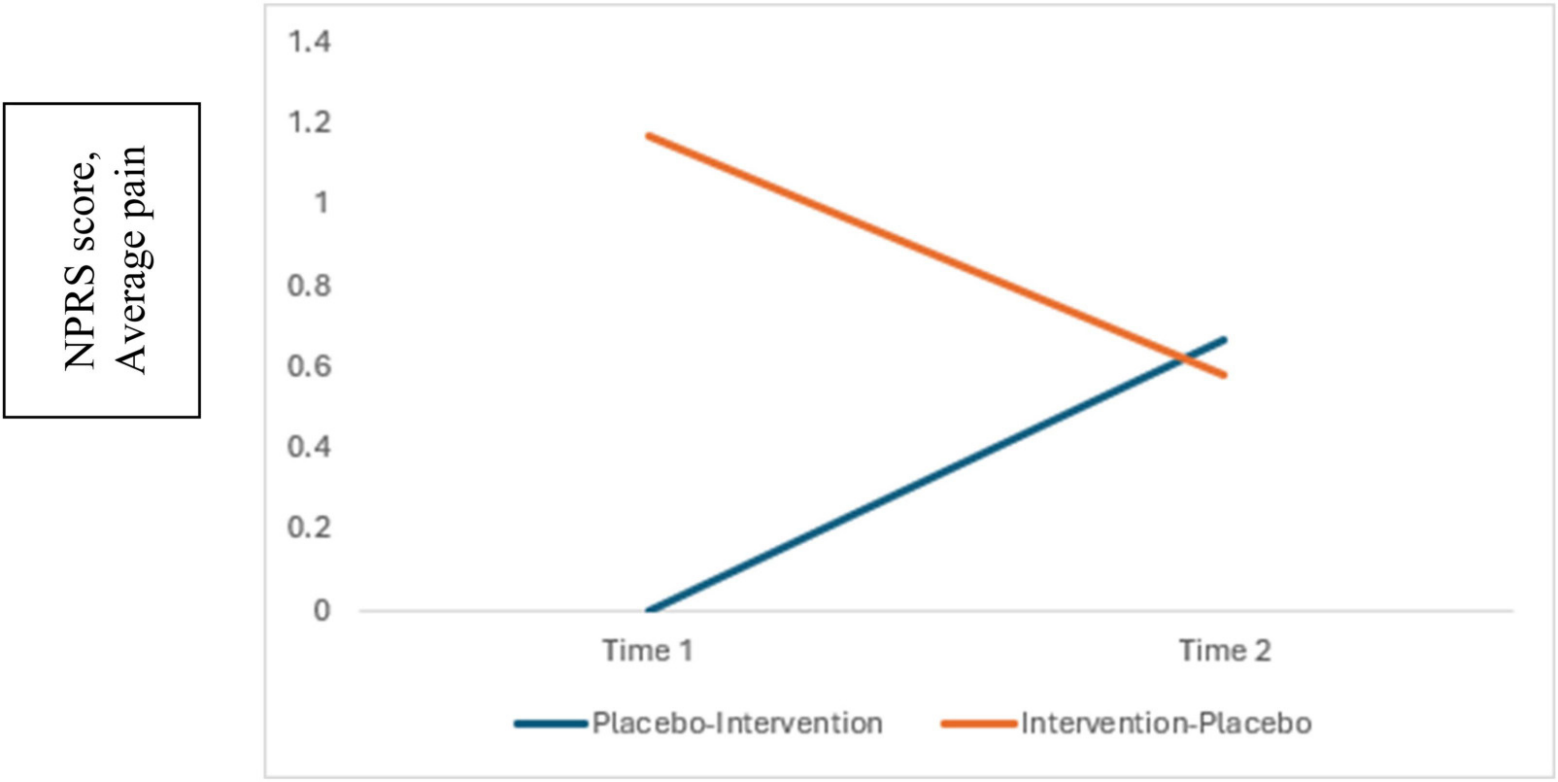
Direct effect of active gel versus placebo gel.

In this crossover study, no significant first-order carryover effects [theta: *t*(22) = −0.24, *p* = 0.816] or second-order carryover effects [lambda: *t*(22) = −1.01, *p* = 0.322] were detected (see Table 4). The change in mean scores from baseline to Day 1 was not significant for the active gel (−0.6 ± 1.4) or the placebo gel (−0.2 ± 1.3), and the difference between the two treatments was not significant (*p* = 0.31). The change in mean scores from baseline to Day 3 for the active treatment was statistically significant (−0.9 ± 1.6, *t* = 2.74, *p* = 0.012), but not for the placebo (−0.1 ± 1.6, *t* = 0.44, *p* = 0.335). The difference between the two treatments (direct treatment effect) trended toward statistical significance [tau: *t*(22) = 1.51, *p* = 0.073, see Figure 3].

Binary outcomes: Twelve (50%) participants improved with the active gel, with an average reduction in NPRS by Day 3 of 32%. Five achieved a ≥30% reduction and three achieved a ≥50% reduction. By contrast, 11 (44%) improved with the placebo gel, with an average reduction in NPRS of 30%, and six achieving a reduction of ≥30%, but none achieving a reduction of ≥50%. Table 5 shows NNT calculations. While the NNT for ≥30% reduction favored the placebo (NNT = 27), the NNT for ≥50% reduction favored the active gel at 8.

**Table 5:** Numbers needed to treat for active gel for 30% and 50% reduction in average pain, day 1 and day 3.

Outcome	Active treatment	*n*	Placebo	*n*	ARR (%)	95% CI of ARR	NNT	95% CI of NNT
Average pain, Day 1								
30% reduction	5	24	2	25	12.83	−6.59% to 32.25%	7.8	>3.1
50% reduction	0	24	0	25	0	–	–	–
Average pain, Day 3								
30% reduction	5	24	6	25	−3.7	−26.5% to 20.16%	−27	>5
50% reduction	3	24	0	25	12.5	−0.74% to 22.97%	8.0	>3.9

Of the 12 improving with the active gel, 9 (67%) also improved by the end of Day 1, and their response at Day 1 (mean of 0.6-point reduction from baseline NPRS for Average Pain) was approximately two-thirds of that achieved by the end of Day 3. Five (37%) also obtained >30% reduction in their NPRS by the end of Day 1, also for an NNT of 8. Only one patient improved on Day 1, which was not maintained to Day 3.

#### 3.3.2 Secondary outcomes

##### 3.3.2.1 Safety

All 25 participants were evaluable for safety. Nineteen AEs occurred, with no SAE. Three AEs were related to the study medication, one of which was Grade 3 (increased numbness at site of application). Reported at the End-of-Treatment visit, it had resolved by the next day, and occurred with the placebo gel.

Four participants reported mild skin irritation, 1–2 out of 7 on the SIS. Three cases occurred with the old formulation, and were observed with both the placebo and active gels.

Six participants had clinically significant changes on the physical exam at the End-of-Study visit, including one neurological change (loss of patellar tendon reflexes). One participant was found to be hypertensive and sent to the ED. Another participant developed abnormal liver function tests that had resolved on retesting 1 month later and deemed unrelated. After 3 days of the active gel, 14 (58%) had higher serum copper levels than at baseline, on average <1 µmol/L. No new increases of copper level above the reference range were observed.

##### 3.3.2.2 Secondary efficacy outcomes

###### 3.3.2.2.1 Change in worst pain NPRS score from baseline to day 1 and day 3

A significant first-order carryover effect for worst pain was noted, with the baseline increasing from Visit 2 to Visit 4, averaged over placebo/intervention [*t*(22) = 2.40, *p* = 0.025]. Seven (29%) participants improved with the active gel and 5 (20%) with the placebo. The average improvement was small (−0.6 vs. −0.3), and a significant direct effect was not found [*t*(22) = −1.10, *p* = 0.283]. Change in NPSI scores were noted from baseline to Day 1 and Day 3.

The average total NPSI score before active gel usage was 35 ± 20. The average change in the total NPSI after 3 days of the active gel usage was 8 ± 14. No statistically significant direct effects were found for NPSI total score or its subscales. Participants with above-average total NPSI score before active treatment were almost twice as likely to respond to the active gel (OR 1.96), although this was not statistically significant (*p* = 0.42). Participants with a total NPSI score greater than the median score (30) were four times more likely to have a reduction from baseline for NPRS score with the active gel, although this estimate was not significant (*p* = 0.11).

There was a trend for the NPSI single-item subscale for burning pain to improve with the active treatment [*t*(22) = −1.94, *p* = 0.067]; however, participants with moderate scores (4–6) for burning pain were more likely to respond than those with severe pain.

#### 3.3.3 *Post hoc* analysis: patients who screened positive on DN4

When participants with DN4 scores less than 4 (*n* = 8) were excluded, improvement in NPRS scores from baseline was significantly greater with the active treatment than control on both Day 1, *t*(14) = −2.19, *p* = 0.046, and Day 3, *t*(14) = −2.17, *p* = 0.047 (see Supplementary data).

## 4 Discussion

### 4.1 Main findings

The aim of this pilot crossover study was to formally evaluate anecdotal reports of the safety and efficacy of topical RM191A for chronic peripheral NPP not responding to other treatments. Consistent with anecdotal reports, topical RM191A had significant analgesic activity when applied four times a day for 3 days, with an average reduction in the average pain NPRS scores of almost 1 out of 10. Although this is less than the MCID of 1.5 points for chronic pain (17), an MCID for NPP has not been established. The placebo had little effect on NPRS, and the difference between the two treatments approached statistical significance. The NNT of 8 for a 50% reduction in NPRS of average pain by Day 3 is lower than that reported for topical lidocaine 5% plasters (NNT = 14.5) and is similar to oral duloxetine (NNT = 7.4) and gabapentinoids (NNT = 8.9) (3). Consistent with the anecdotal reports of fast-onset analgesia, improvements were often seen in the first 24 h of application, with three-quarters of participants improving by the end of the first day. One in six participants obtained >30% reduction in NPRS by end of Day 1 using the active gel.

The NPSI is reported to be sensitive to treatment changes and able to predict treatment outcomes (12), but in this study, no statistically significant direct effects were found for the NPSI total score or subscales overall. The change in the total NPSI with the active gel reached the reported MCID of 7–8 (20). Participants with baseline total NPSI score ≥30 responded better to the active gel, suggesting that this threshold may be worth considering as an inclusion criterion in future studies.

Few AEs were attributable to the study drug, and there were no SAEs. The gel was well tolerated once microcellulose was removed from the vehicle. As a result, the on-treatment dropout rate was lower than expected.

An accurate diagnosis is important when testing a new treatment for neuropathic pain, but doing so remains challenging. Options include clinical evaluation (history and physical exam), screening questionnaires, quantitative sensory testing, and/or more invasive methods (neurophysiology, skin biopsy, corneal confocal microscopy, and/or functional neuroimaging). A joint task force of the European Academy of Neurology, European Pain Federation, and Neuropathic Pain Special Interest Group of the International Association for the Study of Pain recently reviewed the evidence on the diagnostic accuracy of these different methods (21). The task force concluded that the evidence base was weak, but screening questionnaires like DN4 and Leeds Assessment of Neuropathic Symptoms and Signs (LANSS) were strongly recommended. This was on account of their relatively high diagnostic accuracy (low positive predictive value but high negative predictive value) when compared to clinical examination, with or without supporting diagnostic tests. Historically, DN4 has been favored over LANSS, as it is quicker and easier to administer (22). PainDETECT only received a weak recommendation, while screening questionnaires lack the undesirable effects and high resources needed for confirmatory tests such as nerve conduction studies/EMG, skin biopsies, and functional neuroimaging.

In this study, we found only fair agreement between the DN4 score and the IASP diagnostic grade, with almost one-in-three of those with probable/certain NPP screening negative on DN4. Those with a DN4 score <4 responded less well to RM191A. When these patients were excluded, the active gel was significantly more effective than the placebo, with a large effect size. A negative DN4 screen should be an exclusion criterion for future studies on RM191A.

The mechanism of action of RM191A is not yet fully understood. The vehicle does not contain menthol or any other known cooling agents which may be effective for NPP (23). *In vitro* and *in vivo* studies have shown RM191A to have potent antioxidant, anti-inflammatory, and immunomodulatory activities. Reactive oxygen species (ROS) and reactive nitrogen species (RNS), particularly the free-radical peroxynitrite, have been implicated in the development of central sensitization and chronic pain (1, 15, 24–27). RM191A is known to downregulate a number of inflammatory cytokines, including TNF-α and interleukins IL-1β and IL-6, and to also downregulate several key biomarkers of pain, including IL-18, brain-derived neurotrophic factor, and serotonin receptor (5HT_2a_). *In vivo* work has also demonstrated RM191A's ability to meaningfully reduce the influx of leukocytes and mast cells, and subsequently reduce the inflammatory response (5). We are currently undertaking preclinical research on its mechanism of action. Preliminary results (unpublished) have shown analgesic activity in a mouse sciatic nerve ligation model of chronic pain.

### 4.2 Limitations

The study reached its recruitment target but took more than twice as long as expected. This was partly because prevalence of NPP in the clinic was lower than expected, while stay-in-place orders during the COVID-19 pandemic also played a role. The effect of 3 days' application of RM191A on NPRS score was less than anticipated, leaving the study underpowered and the point estimates imprecise. It was noteworthy that while the placebo gel had only a small effect on the NPRS, placebo responses of 30% or more were reported by almost half of the participants. This is consistent with reports of placebo (and nocebo) effects with topical analgesics (28). Including patients with DN4 scores <4 is likely to have reduced the efficacy of the active gel.

### 4.3 Generalizability of the results

There were equal numbers of men and women in this study. The median age of participants was almost 70 years, making the results applicable to older patients, in whom drug side effects and polypharmacy need to be avoided. The population was heterogeneous for the underlying neurological diagnosis, although the numbers in each disease type were too small to assess its relative effectiveness. The study was undertaken in a hospital-based pain clinic, which is tertiary level care for patients referred from primary or specialist care for distressing, disabling pain not responding to other treatments; the results may be different in a non-tertiary setting. Application was only for 3 days, so the outcomes of long-term use are unknown. Future studies should focus on longer-term administration (1–2 weeks) and on specific NPP entities (e.g., diabetic neuropathy, chemotherapy-induced peripheral neuropathy, postherpetic neuralgia), ideally in the primary care or specialist setting, excluding patients who do not have probable or definite neuropathic pain and/or DN4 scores <4.

### 4.4 Interpretation/conclusions

In this small pilot study, short-term administration of RM191A, a potent SOD1 and SOD3 mimetic, was safe and demonstrated modest analgesic activity after 3 days, application in patients with moderate-to-severe chronic peripheral NPP. The study was underpowered to demonstrate superiority compared with placebo, but results approached significance. The duration of application of the active gel may have been insufficient for the full effect of the active gel to be observed. Based on the results from randomization in Treatment Period 1 alone, a total sample size of 80–110 participants would be required, with a treatment duration of 1–2 weeks. Preclinical studies are currently underway to elucidate the mechanism of analgesic action.

## Data Availability

The raw data supporting the conclusions of this article will be made available by the authors without undue reservation.
